# Normalization of Time-Intensity Curves for Quantification of Foot Perfusion Using Near-Infrared Fluorescence Imaging With Indocyanine Green

**DOI:** 10.1177/15266028221081085

**Published:** 2022-03-03

**Authors:** Pim Van Den Hoven, Floris Tange, Jurrian Van Der Valk, Nikolaj Nerup, Hein Putter, Catharina Van Rijswijk, Jan Van Schaik, Abbey Schepers, Alexander Vahrmeijer, Jaap Hamming, Joost Van Der Vorst

**Affiliations:** 1 Department of Surgery, Leiden University Medical Center, Leiden, The Netherlands; 2 Department of Surgical Gastroenterology, Copenhagen University Hospital, Copenhagen, Denmark; 3 Department of Medical Statistics, Leiden University Medical Center, Leiden, The Netherlands; 4 Department of Interventional Radiology, Leiden University Medical Center, Leiden, The Netherlands

**Keywords:** near-infrared fluorescence imaging, indocyanine green, perfusion, lower extremity arterial disease, peripheral artery disease, normalization, quantification

## Abstract

**Purpose:**

Near-infrared (NIR) fluorescence imaging using indocyanine green (ICG) is gaining popularity for the quantification of tissue perfusion, including foot perfusion in patients with lower extremity arterial disease (LEAD). However, the absolute fluorescence intensity is influenced by patient—and system-related factors limiting reliable and valid quantification. To enhance the quality of quantitative perfusion assessment using ICG NIR fluorescence imaging, normalization of the measured time-intensity curves seems useful.

**Materials and Methods:**

In this cohort study, the effect of normalization on 2 aspects of ICG NIR fluorescence imaging in assessment of foot perfusion was measured: the repeatability and the region selection. Following intravenous administration of ICG, the NIR fluorescence intensity in both feet was recorded for 10 mins using the Quest Spectrum platform^®^. The effect of normalization on repeatability was measured in the nontreated foot in patients undergoing unilateral revascularization preprocedural and postprocedural (repeatability group). The effect of normalization on region selection was performed in patients without LEAD (region selection group). Absolute and normalized time-intensity curves were compared.

**Results:**

Successful ICG NIR fluorescence imaging was performed in 54 patients (repeatability group, n = 38; region selection group, n = 16). For the repeatability group, normalization of the time-intensity curves displayed a comparable inflow pattern for repeated measurements. For the region selection group, the maximum fluorescence intensity (Imax) demonstrated significant differences between the 3 measured regions of the foot (*P* = .002). Following normalization, the time-intensity curves in both feet were comparable for all 3 regions.

**Conclusion:**

This study shows the effect of normalization of time-intensity curves on both the repeatability and region selection in ICG NIR fluorescence imaging. The significant difference between absolute parameters in various regions of the foot demonstrates the limitation of absolute intensity in interpreting tissue perfusion. Therefore, normalization and standardization of camera settings are essential steps toward reliable and valid quantification of tissue perfusion using ICG NIR fluorescence imaging.

## Introduction

Near-infrared (NIR) fluorescence imaging has been used as an imaging modality for various indications, including tumor visualization, identification of vital structures, and assessment of tissue perfusion.^1–4^ For the assessment of tissue perfusion, NIR fluorescence imaging has shown potential value in various fields, including vascular, gastrointestinal, and reconstructive surgery.^2,5,6^ However, there are several factors that influence the stability of the fluorescence intensity which compromise the reliability and validity of the technique, precluding broad application in clinical practice.^3,5^ Fluorescence imaging in the NIR light spectrum (700-900 nm) has the advantage of high tissue penetration and low autofluorescence, allowing for clear visualization of a fluorophore with an emission peak in the NIR spectrum.^7,8^ For the assessment of tissue perfusion, the most utilized fluorophore in NIR fluorescence imaging is indocyanine green (ICG). The feasibility of ICG for the assessment of tissue perfusion is explained by the binding to plasma proteins combined with a short half-life due to rapid clearance by the liver.^9^ Following intravenous administration of ICG, information about tissue perfusion can be obtained using either a qualitative or quantitative interpretation of the fluorescence intensity. Applications of qualitative interpretation include the assessment of skin viability in reconstructive surgery and the evaluation of bowl perfusion in gastrointestinal surgery.^10,11^ However, the qualitative and therefore subjective interpretation of the fluorescence intensity leads to different surgical outcomes and impedes comparison between studies.^12^ Quantitative assessment of perfusion focuses on describing the fluorescence intensity change over time in a region of interest (ROI), representing the dynamic properties of blood flow.^13^ Although the benefits of reliable quantitative analysis are evident, several factors influence the stability of the measured fluorescence intensity.^3,8^ These factors are either related to the patient or the camera system. Patient-related factors include skin type, edema, ICG concentration, and the presence of ulcers.^14,15^ The change in tissue properties can lead to an alteration in excitation energy and quantum yield, influencing the measured fluorescence intensity.^8^ Furthermore, the configuration of the camera system affects the measured intensity in several ways, including distance and angle to the target area, the distribution of illumination amongst the field of view, and the optical settings comprising the exposure time and gain.^16,17^ This abundance of influencing factors raises questions as to whether analysis of the absolute intensity is appropriate for reliable assessment of tissue perfusion.^3,5^ An emerging analyzing method for the assessment of tissue perfusion that adjusts for absolute intensity is normalization.^18^ Normalization sets the maximum fluorescence intensity at 100% and displays the fluorescence intensity over time as a percentual change. It is hypothesized that this method decreases the influence of aforementioned influencing factors and allows for more reliable and valid quantification. Therefore, the aim of this study was to investigate the influence of normalization on repeatability and region selection of ICG NIR fluorescence imaging for assessment of foot perfusion.

## Materials and Methods

This cohort study was approved by the Medical Research and Ethics Committee of the Leiden University Medical Center and registered in the Dutch Trial Register (#NL7531). Patients undergoing unilateral revascularization procedures and non-lower extremity arterial disease (LEAD) control patients were included. Patients were included in a single academic hospital in the Netherlands from December 2018 until April 2021. Patients were excluded based on contraindications to ICG: allergy or hypersensitivity to ICG or (sodium) iodide; hyperthyroidism, autonomous thyroid adenoma, pregnancy, kidney failure (estimated glomerular filtration rate [eGFR] < 45), or severe liver failure. Informed consent was obtained from all individual participants included in the study.

### ICG NIR Fluorescence Imaging

ICG NIR fluorescence imaging measurements were performed using the Quest Spectrum Platform (Quest Medical Imaging, Middenmeer, The Netherlands). This system consists of an LED laser combined with a camera measuring light in the visible and NIR light spectrum (700-900 nm). All patients underwent ankle-brachial index (ABI)—and toe pressure (TP) measurements prior to the ICG NIR fluorescence imaging measurement. Upon ICG (VERDYE 25 mg, Diagnostic Green GmbH, Aschheim-Dornach, Germany) administration, the camera registered the NIR fluorescence intensity change over time in both feet for 10 mins. All measurements were performed with the patient in a supine position in a darkened room. The camera was placed at approximately 50 cm of the foot, perpendicular to the dorsum of the foot. All videos were recorded using an exposure time of 145 ms and a gain of 22 db.

### Repeatability Group

The effect of normalization on repeatability was measured in the nontreated foot of patients undergoing unilateral revascularization. It was hypothesized that the time-intensity curves in the nontreated contralateral foot remained unchanged. A subset of this group was described in an earlier study in which only normalized data were used.^19^ ICG NIR fluorescence imaging was performed before—and after the procedure (<3 days), and patients were administered an intravenous bolus injection of 0.1 mg/kg ICG. Three ROIs were analyzed: (1) the dorsum of the foot, (2) the forefoot, and (3) the hallux.

### Region Selection Group

The effect of normalization on region selection was measured in non-LEAD control patients because this group is most likely to display a homogenous perfusion pattern in various regions of the foot. This group consisted of patients undergoing liver metastasectomy who were administered ICG intravenously 1 day before surgery. In this group, a bolus injection of 10 mg ICG was administered intravenously as part of the noninvestigational treatment protocol. Three regions were selected based on differences in camera distance and angle of the surface area to the camera. These regions included: (1) the hallux, (2) the first ray of the foot, and (3) the lateral foot.

### Data Analysis

The Quest Research Framework^®^ (Quest Medical Imaging, Middenmeer, the Netherlands) software was used for the quantification of the measured NIR fluorescence intensity. Following manual selection of an ROI, the software creates a curve of the fluorescence intensity change over time. The measured fluorescence intensity is displayed as arbitrary units (a.u.). When normalization is applied, the software sets the maximum fluorescence intensity in the selected ROI at 100% and displays the fluorescence intensity over time as a percentual change of this maximum fluorescence. The absolute time-intensity curves and normalized time-intensity curves with the extracted parameters are displayed in Supplementary Figure 1 and Supplementary Table 1. After normalization, absolute parameters including ingress and ingress rate are depleted. The normalized slope for the ingress and egress are defined as percentage per second. A tracker was used to ensure that the ROI was synchronized with foot movement, and baseline subtraction was applied. Starting time of the time-intensity curves was defined as an increase of 1 a.u. for the absolute time-intensity curves and 1% for the normalized time-intensity curves. Statistical analyses were performed using IBM SPSS Statistics 25 (IBM Corp. Released 2017. IBM SPSS Statistics for Windows, Version 25.0. Armonk, NY, USA: IBM Corp.). Parameters in the repeatability group were compared with the Wilcoxon rank-sign test for paired analyses. Results for the region selection group were compared using the Kruskal-Wallis test.

## Results

Indocyanine green NIR fluorescence imaging was successfully performed in 54 patients. The patient characteristics for both the repeatability group and region selection group are displayed in Table 1. In the repeatability group, consisting of 38 patients, the mean age was 70.9 years with a standard deviation (SD) of 7.0. The mean ABI in this group was 0.89 (SD 0.30) with a mean TP of 76 mm Hg (SD 30). The 16 patients (32 limbs) in the region-selection group displayed a mean age of 66.6 years (SD 12.3) with a mean ABI of 1.11 (SD 0.10) and a mean TP of 106 mm Hg (SD 22).

**Table 1.** Patient Characteristics.

	Repeatability group (n = 38)	Region selection group (n = 16)
Age in years (SD)	70.9 (7.0)	66.6 (12.3)
Female, n (%)	17 (44.7)	3 (18.8)
Diabetes, n (%)	12 (31.6)	3 (18.8)
Hypertension, n (%)	29 (76.3)	7 (43.8)
Active smoking, n (%)	8 (21.1)	1 (6.3)
History of smoking, n (%)	35 (92.1)	10 (62.5)
Mean baseline ABI (SD)	0.89 (0.30)	1.11 (0.10)
Mean baseline TP (SD)	76 (30)	106 (22)

Abbreviations: ABI, ankle-brachial index; TP, toe pressure.

### Repeatability Group

**Figure 1.**
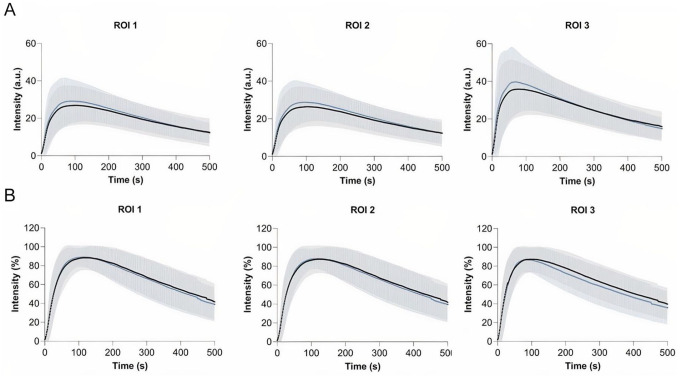
Repeatability group—time-intensity curves for the dorsum of the foot (ROI 1), the forefoot (ROI 2) and the hallux (ROI 3) before- (black line) and after the revascularization procedure (blue line). Results are displayed as mean (bold line) with one standard deviation confidence bands: (A) absolute time-intensity curves and (B) normalized time-intensity curves. ROI, region of interest.

For the repeatability group, no significant differences were found for the ABI and TP preprocedural and postprocedural (ABI: 0.89 vs 0.86, *P* = .806; TP: 76 vs 72, *P* = .466). The results on repeated measurements for both the absolute and normalized time-intensity curves in the repeatability group are displayed in Figure 1. For the absolute time-intensity curves, an increase in maximum fluorescence intensity (Imax) is seen for all 3 ROIs for the repeated measurements. Furthermore, the absolute time-intensity curves in ROI 3 display a wider distribution compared to the other ROIs. After normalization, the time-intensity curves display a similar distribution amongst all 3 ROIs. Furthermore, the inflow pattern is comparable for repeated measurements. The results on quantification of the time-intensity curves for the repeatability group are depicted in Table 2. Except for the area under the curve (AUC) ingress in ROI 3, no statistical differences were found for all measured parameters in all ROIs. Although not significant, absolute parameters including the Imax, ingress rate, and slope were higher for the repeated measurement. For the ingress rate in ROI 1 and 2, the repeated measurement displayed a 50% increase (ROI1: 0.4 vs 0.6, *P* = .587, ROI 2: 0.4 vs 0.6, *P* = .404). After normalization, the slope in all ROIs were comparable (ROI 1: 3.4 vs 3.5, *P* = .983, ROI 2: 3.4 vs 3.4, *P* = .936, ROI 3: 0.8 vs 1.0, *P* = .502).

**Table 2.** Repeatability Group—ICG NIR Fluorescence Imaging Outcome.

N = 38 (38 limbs)	Pre	Post	*P*						
	Mean ± SD	Mean ± SD							
ABI	0.89 ± 0.30	0.86 ± 0.33	.806						
TP	76 ± 30	72 ± 27	.466						
ICG NIR fluorescence parameters	ROI 1			ROI 2			ROI 3		
	Pre	Post	*P*	Pre	Post	*P*	Pre	Post	*P*
	Mean ± SD	Mean ± SD		Mean ± SD	Mean ± SD		Mean ± SD	Mean ± SD	
Tmax	111.8 ± 68.1	102.8 ± 61.3	.346	115.1 ± 67.8	103.8 ± 60.9	.218	90.1 ±58.1	75.0 ± 48.4	.177
AUC10	47.6 ± 2.3	47.4 ± 1.9	.733	47.5 ± 2.8	47.3 ± 2.0	.538	47.6 ± 2.7	47.7 ± 4.6	.922
AUC ingress	71.6 ± 4.9	70.6 ± 5.6	.538	70.9 ± 5.5	69.5 ± 5.8	.438	70.0 ± 5.6	66.5 ± 7.9	.028
AUC egress 60	95.2 ± 4.5	94.8 ± 4.1	.879	95.0 ± 4.9	94.8 ± 4.8	.567	93.5 ± 5.8	91.8 ± 7.8	.331
AUC egress 120	90.2 ± 6.8	89.5 ± 6.6	.617	89.7 ± 7.5	89.3 ± 6.9	.500	87.8 ± 8.5	85.4 ± 10.2	.242
AUC egress 180	84.9 ± 8.4	83.9 ± 8.3	.645	84.3 ± 9.0	83.5 ± 8.5	.521	82.4 ± 9.9	79.8 ± 11.5	.172
AUC egress 240	79.5 ± 9.4	78.5 ± 9.5	.626	79.2 ± 10.0	78.4 ± 9.7	.572	77.6 ± 10.6	74.4 ± 12.2	.177
AUC egress 300	74.6 ± 10.2	69.9 ± 12.8	.623	73.9 ± 10.7	73.2 ± 10.5	.578	72.6 ± 10.9	69.9 ± 12.8	.172
Imax	30.0 ± 10.8	32.9 ± 13.5	.286	29.7 ± 10.8	32.9 ± 12.6	.204	41.2 ± 16.7	47.1 ± 22.2	.187
Ingress rate	0.4 ± 0.5	0.6 ± 0.8	.587	0.4 ± 0.5	0.6 ± 0.7	.404	0.8 ± 0.9	1.2 ± 1.6	.150
Absolute slope ingress	1.1 ± 0.9	1.4 ± 1.5	.528	1.1 ± 0.8	1.4 ± 1.4	.293	1.9 ± 1.8	2.7 ± 3.0	.158
Absolute slope egress	0.3 ± 0.1	0.3 ± 0.2	.833	0.2 ± 0.1	0.3 ± 0.1	.350	0.3 ± 0.2	0.5 ± 0.5	.133
Normalized slope ingress	3.4 ± 2.0	3.5 ± 2.4	.983	3.4 ± 2.0	3.4 ± 2.3	.936	4.1 ± 2.4	4.5 ± 2.9	.656
Normalized slope egress	0.8 ± 0.4	0.8 ± 0.5	.547	0.8 ± 0.4	0.7 ± 0.3	.313	0.8 ± 0.4	1.0 ± 0.7	.502

Abbreviations: ABI, ankle-brachial index; AUC, area under the curve; ICG, indocyanine green; NIR, near-infrared; ROI, region of interest; TP, toe pressure.

### Region Selection Group

**Figure 2.**
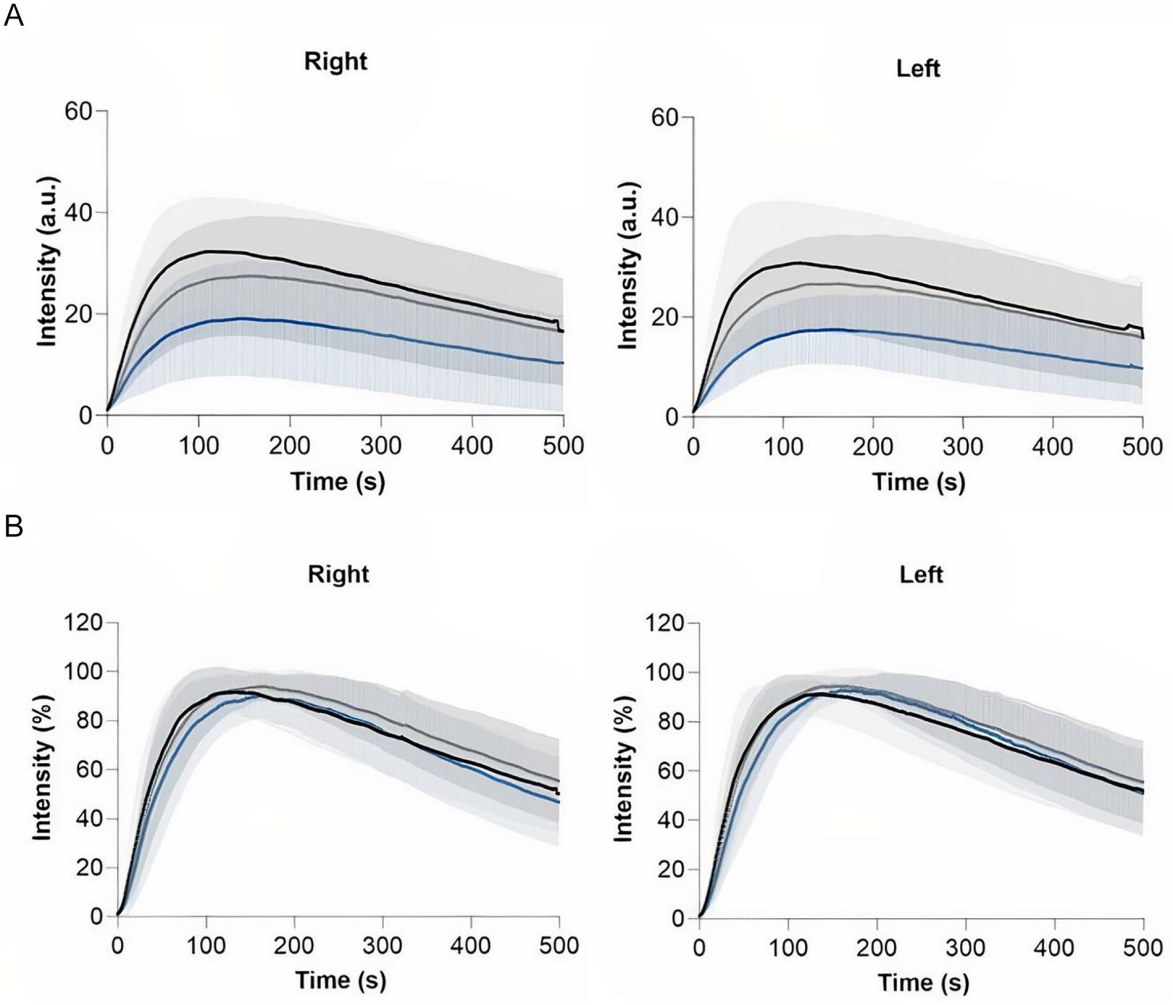
Region selection group—time-intensity curves for the right and left foot displaying the hallux region (black line), first ray region (gray line) and lateral foot region (blue line). Results are displayed as mean (bold line) with one standard deviation confidence bands: (A) absolute time-intensity curves and (B) normalized time-intensity curves.

**Figure 3.**
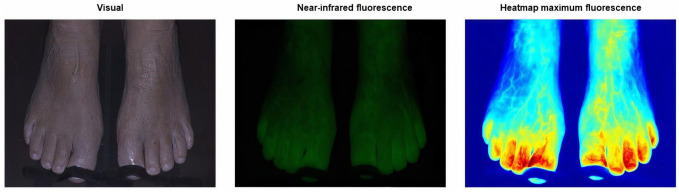
Output of the Quest Research Framework showing the visual light (left), near-infrared fluorescence (middle) and heatmap of the maximum fluorescence intensity (right) of a 70-year-old female control patient. The heatmap illustrates the diminished observed maximum fluorescence intensity found on the lateral side of the foot.

The absolute and normalized time-intensity curves for the region selection group are visualized in Figure 2. Results for the right and left foot are displayed separately. For the absolute time-intensity curves, there is a clear discrepancy for the measured maximum fluorescence intensity between the 3 ROIs. The lowest maximum intensity is seen in ROI 3, that is, the lateral foot, which is visualized in Figure 3. Furthermore, there is a wide distribution amongst all 3 measured ROIs. After normalization, the patterns observed in both the right and left foot are comparable for all 3 ROIs. The extracted parameters for the region selection group are displayed in Table 3. For the fixed parameters, no significant differences were found for all 3 ROIs in both feet. For the absolute parameters, a statistical significance was seen for the Imax, ingress rate, and slope ingress in the right as well as the left foot (right: *P* = .002, .015, .005; left: *P* = .006, .011, .037, respectively). After normalization, a significant difference was seen for the slope egress in both feet (right: *P* < .001, left: *P* < .001). The normalized ingress slope was comparable for all ROIs (right: *P* = .408, left *P* = .921).

**Table 3.** Region Selection Group—ICG NIR Fluorescence Imaging Outcome.

N = 16 (32 limbs)	Right foot			*P*	Left foot			*P*
	First ray	Lateral foot	Hallux		First ray	Lateral foot	Hallux	
ICG NIR fluorescence parameters	Mean ± SD	Mean ± SD	Mean ± SD		Mean ± SD	Mean ± SD	Mean ± SD	
Tmax	157.7 ± 69.4	161.7 ± 69.8	126.2 ± 64.6	.353	157.5 ± 74.9	153.5 ± 72.6	124.3 ± 73.5	.341
AUC10	50.0 ± 5.0	48.7 ± 4.7	49.8 ± 5.2	.663	48.9 ± 6.3	49.3 ± 6.0	50.9 ± 6.4	.547
AUC ingress	72.5 ± 5.3	68.7 ± 4.7	69.8 ± 5.7	.147	72.2 ± 4.0	67.8 ± 5.0	69.5 ± 4.2	.039
AUC egress 60	96.9 ± 2.0	95.8 ± 1.9	96.2 ± 2.7	.161	97.1 ± 1.9	95.0 ± 4.1	96.0 ± 2.8	.140
AUC egress 120	93.3 ± 3.1	91.9 ± 3.2	91.8 ± 4.7	.470	93.6 ± 2.5	90.7 ± 4.9	91.8 ± 4.8	.287
AUC egress 180	89.1 ± 4.5	87.1 ± 4.6	87.4 ± 6.1	.541	89.4 ± 3.5	85.6 ± 5.5	87.5 ± 6.2	.169
AUC egress 240	84.9 ± 5.6	81.6 ± 5.0	83.0 ± 7.1	.270	85.1 ± 4.4	79.9 ± 5.8	83.3 ± 7.1	.061
AUC egress 300	80.3 ± 6.0	76.9 ± 5.7	78.2 ± 7.6	.388	80.3 ± 5.0	74.7 ± 6.3	78.5 ± 7.4	.065
Imax	28.4 ± 18.8	18.9 ± 7.8	33.7 ± 14.3	.002	29.3 ± 12.8	21.1 ± 12.1	35.2 ± 11.8	.006
Ingress rate	0.2 ± 0.1	0.1 ± 0.1	0.4 ± 0.4	.015	0.2 ± 0.1	0.2 ± 0.1	0.4 ± 0.3	.011
Absolute slope ingress	0.7 ± 0.4	0.4 ± 0.2	1.0 ± 0.7	.005	0.7 ± 0.4	0.6 ± 0.4	1.0 ± 0.7	.037
Absolute slope egress	0.2 ± 0.1	0.2 ± 0.1	0.2 ± 0.1	.288	0.2 ± 0.1	0.2 ± 0.1	0.2 ± 0.1	.104
Normalized slope ingress	2.4 ± 0.8	2.2 ± 0.6	2.8 ± 1.3	.408	2.4 ± 0.8	2.7 ± 1.5	2.9 ± 1.9	.921
Normalized slope egress	0.7 ± 0.3	1.2 ± 0.5	0.6 ± 0.2	<.001	0.6 ± 0.3	1.2 ± 0.5	0.6 ± 0.2	<.001

Abbreviations: AUC, area under the curve; ICG, indocyanine green; NIR, near-infrared.

## Discussion

This study demonstrates the effect of normalization of time-intensity curves on both the repeatability and region selection in the quantification of tissue perfusion using ICG NIR fluorescence imaging. Concerning repeatability, time-intensity curves display a more similar pattern following normalization. Although not significant, absolute parameters including ingress rate and slope varied between measurements and displayed a wider distribution. These findings suggest that absolute parameters are less reliable and more susceptive to fluctuations on repeated measurements. The repeatability of ICG NIR fluorescence imaging for assessment of tissue perfusion in patients with LEAD was described in one earlier study.^20^ This study found time-intensity curves to be repeatable and focused on time as well as absolute parameters. However, repeated measurements were performed in the same setting by the same investigator, thus reducing the impact of influencing factors of measurement setup on the NIR signal. Regarding the region selection, absolute inflow parameters in this study were all significantly different between various areas of the foot. After normalization, the slope ingress was comparable. For the interpretation of tissue perfusion with ICG NIR fluorescence imaging, these are important findings because absolute parameters can thus lead to an incorrect interpretation of actual tissue perfusion. In the search for reliable quantification of tissue perfusion with ICG NIR fluorescence imaging, an abundance of parameters have been studied in various target tissues.^13^ For the quantification of skin perfusion in patients with LEAD, for example, time-related and normalized parameters appear to be superior to measurements of maximum intensity.^21,22^ In reconstructive surgery, a commonly performed analyzing method for tissue quantification is the use of relative parameters.^6^ However, this method does not take into account the camera angle and distance, leading to a misperception of actual perfusion. In gastrointestinal surgery, the effect of normalization on quantification of bowl perfusion was measured in several studies.^3,18,23^ In a series of studies by Nerup et al on gastrointestinal perfusion in porcine models, the normalized slope ingress was significantly correlated with regional blood flow and local lactate levels.^18,23^ Although reliable quantification of tissue perfusion seems to tend toward the use of normalized parameters, several items have to be discussed. First of all, normalization of the time-intensity curves leads to alteration of data, which precludes the use of absolute parameters that can be useful in the prediction of tissue necrosis.^24^ Furthermore, normalization can be unreliable when the measured fluorescence intensity levels are below a certain threshold. Magnification of the signal can then lead to high fluctuations in the percentual change, which is the presumable cause of the significant increase in normalized egress slope in the region selection group in this study. In addressing the effect of normalization on region selection, the cohort in this study consisted of patients without known LEAD who were administered ICG as part of the treatment protocol for liver metastasectomy. By selecting this group of patients, this study avoided the exposure of healthy volunteers to ICG. However, due to comorbidities, there might have been changes in regional circulation of the foot that could have influenced the measured fluorescence intensity. Therefore, future studies on ICG NIR fluorescence imaging for foot perfusion assessment should ideally be performed in a control group that has a significantly lower risk of possible unknown underlying LEAD. Concerning the repeatability group, this study is limited by the small sample size. Besides, measurements were performed on different days postprocedural, which might have led to interpatient variability. Furthermore, changes in hemodynamic status, including blood pressure and pulse could have influenced the measured fluorescence intensity.^25^ Despite these limitations, this study describes a new perspective on assessment of tissue perfusion in the foot using ICG NIR fluorescence imaging. Improving reliability and validity of ICG NIR fluorescence imaging in quantification of tissue perfusion using normalization can promote comparability between studies. To compare standardized quantification methods between studies, it is also of paramount importance to report on the used camera system and—settings, including exposure time and gain. Addressing these aspects in future studies on perfusion assessment using ICG NIR fluorescence imaging is an essential step toward reliable quantification. Whether this quantification will lead to a better understanding of actual inflow and outflow of foot perfusion has yet to be determined. However, reliable quantification will be a crucial factor in the value of future studies on perfusion assessment with ICG NIR fluorescence imaging. Therefore, normalization should be a standard procedure in the analysis of the measured fluorescence intensity in these studies. The valid and reliable assessment of tissue perfusion using ICG NIR fluorescence imaging could then potentially aid in the prediction of clinical outcome following revascularization or in assessing the probability of wound healing.

## Conclusion

This study shows the effect of normalization of time-intensity curves on both the repeatability and region selection for the quantification of foot perfusion using ICG NIR fluorescence imaging. The significant difference between absolute parameters in various regions of the foot demonstrates the limitation of absolute intensity in interpreting tissue perfusion. Therefore, normalization and standardization of camera settings are essential steps toward reliable and valid quantification of tissue perfusion using ICG NIR fluorescence imaging.

## Supplemental Material

sj-docx-1-jet-10.1177_15266028221081085 – Supplemental material for Normalization of Time-Intensity Curves for Quantification of Foot Perfusion Using Near-Infrared Fluorescence Imaging With Indocyanine Green Click here for additional data file. Supplemental material, sj-docx-1-jet-10.1177_15266028221081085 for Normalization of Time-Intensity Curves for Quantification of Foot Perfusion Using Near-Infrared Fluorescence Imaging With Indocyanine Green by Pim Van Den Hoven, Floris Tange, Jurrian Van Der Valk, Nikolaj Nerup, Hein Putter, Catharina Van Rijswijk, Jan Van Schaik, Abbey Schepers, Alexander Vahrmeijer, Jaap Hamming and Joost Van Der Vorst in Journal of Endovascular Therapy

sj-jpg-1-jet-10.1177_15266028221081085 – Supplemental material for Normalization of Time-Intensity Curves for Quantification of Foot Perfusion Using Near-Infrared Fluorescence Imaging With Indocyanine Green Click here for additional data file. Supplemental material, sj-jpg-1-jet-10.1177_15266028221081085 for Normalization of Time-Intensity Curves for Quantification of Foot Perfusion Using Near-Infrared Fluorescence Imaging With Indocyanine Green by Pim Van Den Hoven, Floris Tange, Jurrian Van Der Valk, Nikolaj Nerup, Hein Putter, Catharina Van Rijswijk, Jan Van Schaik, Abbey Schepers, Alexander Vahrmeijer, Jaap Hamming and Joost Van Der Vorst in Journal of Endovascular Therapy
