# Supplementary material for: Normalization of Time-Intensity Curves for Quantification of Foot Perfusion Using Near-Infrared Fluorescence Imaging With Indocyanine Green
Source: J Endovasc Ther. 2022 Mar 3;30(3):364–71. doi: 10.1177/15266028221081085 (PMC10209496; doi:10.1177/15266028221081085)
Supplement: sj-docx-1-jet-10.1177_15266028221081085 – Supplemental material for Normalization of Time-Intensity Curves for Quantification of Foot Perfusion Using Near-Infrared Fluorescence Imaging With Indocyanine Green [file sj-docx-1-jet-10.1177_15266028221081085.docx]

| **Fixed parameters** | **Absolute parameters** | **Normalized parameters** |
| --- | --- | --- |
| Tmax (s) | Imax (a.u.) | Slope ingress (%/s) |
| AUC10 (%) | Ingress rate (a.u./s) | Slope egress (%/s) |
| AUC ingress (%) | Slope ingress (a.u./s) |  |
| AUC egress 60 (%) | Slope egress (a.u./s) |  |
| AUC egress 120 (%) |  |  |
| AUC egress 180 (%) |  |  |
| AUC egress 240 (%) |  |  |
| AUC egress 300 (%) |  |  |

**Supplementary Table 1. ICG NIR fluorescence imaging parameters**

Abbreviations: AUC, area under the curve; a.u., arbitrary unit(s); s, seconds.
