# Supplementary figures and images for: Normalization of Time-Intensity Curves for Quantification of Foot Perfusion Using Near-Infrared Fluorescence Imaging With Indocyanine Green
Source: J Endovasc Ther. 2022 Mar 3;30(3):364–71. doi: 10.1177/15266028221081085 (PMC10209496; doi:10.1177/15266028221081085)

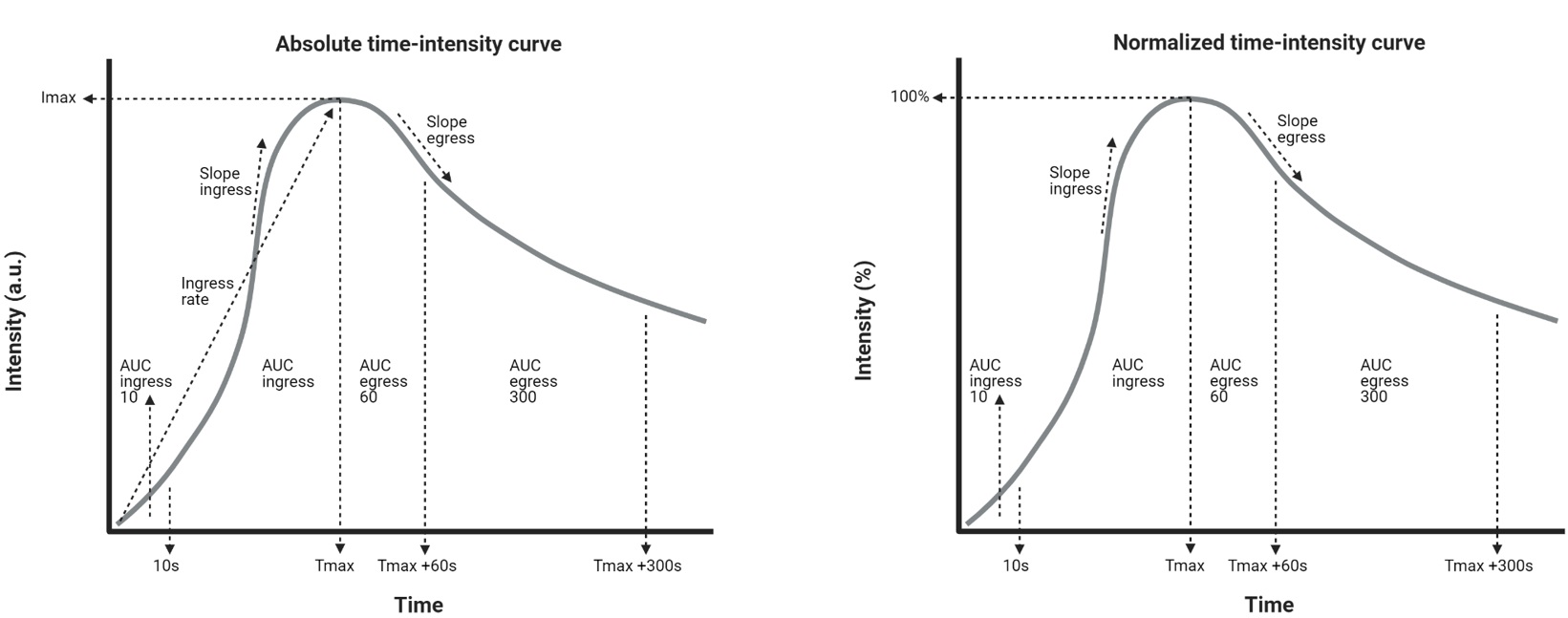

Supplement: sj-jpg-1-jet-10.1177_15266028221081085 – Supplemental material for Normalization of Time-Intensity Curves for Quantification of Foot Perfusion Using Near-Infrared Fluorescence Imaging With Indocyanine Green [file sj-jpg-1-jet-10.1177_15266028221081085.JPG]
